# Factors associated with heartworm preventative use in the golden retriever lifetime study

**DOI:** 10.3389/fvets.2023.1208804

**Published:** 2023-06-09

**Authors:** Lauren Wisnieski, Vina Faulkner, Charles Faulkner

**Affiliations:** Richard A. Gillespie College of Veterinary Medicine, Lincoln Memorial University, Harrogate, TN, United States

**Keywords:** elastic net regression, heartworm disease, prophylactic, bootstrapping, canine, preventative, dog, big data

## Abstract

**Introduction:**

Heartworm disease is preventable with use of heartworm preventatives, but the reported prevalence of heartworm preventative use in the United States is low, some estimates falling around 50% of dogs. However, there are very few estimates of prevalence and its associated factors.

**Methods:**

We aimed to estimate prevalence and evaluate factors, including vaccination status, demographics, lifestyle, physical conditions, medications and supplements, and environment and living conditions, for their association with heartworm preventative use in a large dataset from the Golden Retriever Lifetime Study (*N* = 2,998). Due to the large number of predictors evaluated, we built a bootstrapped elastic net logistic regression model, which is robust to overfitting and multicollinearity. Variables were evaluated by calculating covariate stability (>80%) and statistical significance (*p*<0.02).

**Results:**

In our sample, the prevalence of heartworm use was 39.5%. In our elastic net model, receiving vaccinations (rabies, Bordetella, or any other vaccine), being located in the Southern U.S., being altered, having an infectious disease or ear/ nose/throat system disease diagnosis, being on heartworm preventatives in the past, currently being on tick preventative, having sun exposure in an area with concrete flooring, living in a house with more rooms with carpeted floors, and spending time on hardwood flooring inside were associated with greater odds of heartworm preventative use. Supplementation use and being in the top quartile of height were associated with lower odds of heartworm preventative use.

**Discussion:**

The explanatory factors we identified can be used to improve client communication. In addition, target populations for educational interventions and outreach can be identified. Future studies can validate the findings in a more diverse population of dogs.

## 1. Introduction

Canine heartworm disease (CHD) incidence and geographic range is increasing in the United States (1, 2). From 2013 to 2016, Drake and Wiseman (1) estimated that the relative change in CHD was +15.3% in the US, with highest increases in the Southeast region. Heartworm disease is preventable with the use of prophylactic macrolide medications, which are safe and effective (3). Previous studies have estimated that adherence to the recommended 12-month use of heartworm preventative is low (24.4% for monthly HWD preventatives) (4, 5). One study found that use of heartworm preventatives was estimated to be approximately 74–79% of dogs presenting to a veterinary teaching hospital, however it was reduced to 50% in winter (5). Very few studies have evaluated what factors are associated with heartworm preventative use in dogs. Bir et al. (6) found greater heartworm preventative use among dogs with owners that were male, younger, lived in the South, and had higher socioeconomic status. Gates and Nolan (5) evaluated multiple factors included signalment, presenting complaint, and vaccination status and found that only month of presentation and neuter status were associated with heartworm preventative use. Due to the sparse research on this topic, there is a need to identify additional factors associated with heartworm preventative use, including lifestyle, the strength of the human-animal bond, the environment, and living conditions. Therefore, the objective of this study was to evaluate the association of multiple factors with heartworm preventative use utilizing data available from the Golden Retriever Lifetime Study, which is a large prospective study that aims to identify risk factors for disease in golden retrievers (7). Due to the large number of predictors evaluated, we utilized elastic net regression, which is a regularized regression method that reduces risk of overfitting and multicollinearity (8).

## 2. Materials and methods

The Institutional Review Board at Lincoln Memorial University approved this study (1,068 V.0). All data cleaning and analyses were conducted in Stata version 17.0 (College Station, TX).

### 2.1. Dataset and data cleaning

The Golden Retriever Lifetime study is a large, prospective cohort study that was established in 2012, which investigates risk factors for a variety of health conditions, including the primary endpoints of lymphoma, hemangiosarcoma, high-grade mast cell tumors, and osteosarcoma. Over each dog’s lifetime, owners and veterinarians complete an annual questionnaire and clinical exam and collect samples. All dogs in the study visit their veterinarian yearly to complete a physical exam and sample collection (7). For this analysis, all data from the owner and veterinarian questionnaires were requested, except for diet. The full list of variables screened can be obtained from the Annual Veterinarian Questionnaire at^1^ and the Annual Owner Questionnaire at.^2^ After receiving the data, variables were further screened for feasibility of a potential relationship with heartworm prophylaxis use by our study team. The remaining variables underwent data cleaning. Numerical variables were evaluated for linearity. Variables that violated linearity requirements and that were unable to be transformed (ex. Logarithm transformation) were converted to categorical variables based on quartiles of the original variable. Categorical variables with more than two categories were converted to dummy variables as required for elastic net regression. Variables with a large amount of missing data or lack of variation were removed from the analysis. Complete case analysis was used, so observations with any missing data of the remaining variables were removed. The final list of predictor variables and how they were coded are presented in Table 1.

**Table 1:** Variable names, descriptions, and coding used for a bootstrapped elastic net model with binary outcome variable “heartworm preventative use.”

Category	Variable	Description	Coding
Demographics	Coat color	Dog’s reported coat color	Converted to 4 dummy variables for blonde, dark gold or red, medium gold, and light gold (1 = yes, 0 = no)
	Region	Region of residence reported on the AOQ	Converted to 5 dummy variables for South, Midwest, Mountain, Northeast, and Pacific (1 = yes, 0 = no)
	Sex	Dog’s reported sex status	Converted to 4 dummy variables for male neutered, male intact, female neutered, and female intact (1 = yes, 0 = no)
Lifestyle	Insurance	Dog health insurance status	1 = yes, 0 = no
	Primary activity	Primacy activity/lifestyle	Converted to 1 dummy variables for companion animal (1 = yes, 0 = no)
	Brushes teeth	Dog gets teeth brushed	1 = yes, 0 = no
	Dental treat	Dog gets dental treats	1 = yes, 0 = no
	Dental food	Dog gets dental food	1 = yes, 0 = no
	Mouthwash	Dog gets mouthwash/water additive	1 = yes, 0 = no
	Homegroom frequency	Homegroom >1x a month	1 = yes, 0 = no
	Professional groom	Ever professional groom	1 = yes, 0 = no
	Sun exposure	Dog’s sun exposure duration	1 = 3 or more hours, 0 = less than 3 h
	Sun exposure location	Dog’s sun exposure location	Converted to 3 dummy variables for concrete, grass, and dirt (1 = yes, 0 = no)
	Swim location	Dog’s swim location	Converted to 4 dummy variables for swimming pool, pond/lake, river/stream/agricultural ditch, and ocean (1 = yes, 0 = no)
	Attachment 1	Dogs tends to sit close to or in contact with owner (or others) when owner (or others) are sitting down	Left as numerical scale (1 = never, 2 = seldom, 3 = sometimes, 4 = usually, 5 = always)
	Attachment 2	Dogs tends to nudge, nuzzle, or paw owner (or others) for attention when owner (or others) are sitting down	Left as numerical scale (1 = never, 2 = seldom, 3 = sometimes, 4 = usually, 5 = always)
	Dog activity level	Dog is active, energetic, always on the go	Left as numerical scale (1 = never, 2 = seldom, 3 = sometimes, 4 = usually, 5 = always)
AOQ medications and supplements	Supplements	Supplements given	1 = yes, 0 = no
	Medication	Medications given	1 = yes, 0 = no
	Tick	Dog is on tick preventative	1 = yes, 0 = no
	Prior heartworm	Dog has been on heartworm preventative in past	1 = yes, 0 = no
	Heartworm	Dog is currently on heartworm preventative	1 = yes, 0 = no
Environment and living conditions	Wood flooring	Number of rooms with wood flooring	0, 1, 2…
	Carpeted flooring	Number of rooms with carpeted flooring	0, 1, 2…
	Tile flooring	Number of rooms with tile flooring	0, 1, 2…
	Linoleum flooring	Rooms with linoleum flooring	1 = yes, 0 = no
	Spends most time	Where dog spends most time	Converted to 2 dummy variables for indoors, indoors/outdoors and outdoors (1 = yes, 0 = no)
	Crate	Dog spends time indoors in indoor crate	1 = yes, 0 = no
	Spends time on carpeted flooring	Dog spends time indoors in a room with carpeted flooring	1 = yes, 0 = no
	Spends time on hardwood flooring	Dog spends time indoors in a room with hardwood flooring	1 = yes, 0 = no
	Spends time on tile flooring	Dog spends time indoors in a room with tile flooring	1 = yes, 0 = no
	Spends time on linoleum flooring	Dog spends time indoors in a room with linoleum flooring	1 = yes, 0 = no
	Spends time in room with furniture	Dog spends time indoors in a room with furniture	1 = yes, 0 = no
	Spends time in another room	Dog spends time indoors in another room	1 = yes, 0 = no
	Kennel	Dog spends time in kennel	1 = yes, 0 = no
	Fenced area	Dog spends time in fenced area	1 = yes, 0 = no
	Chain or lead	Dog spends time on a chain or lead	1 = yes, 0 = no
	Poison control	Number of poison control or veterinarian calls due to dog ingesting poison or other hazardous material in the past 12 months	Converted to 1 dummy variable (0 = never, 1 = at least once)
	Smoke	Dog exposed to secondhand smoke in the last 12 months	1 = yes, 0 = no
	Neighborhood type	Primary neighborhood type dog has lived in the past 12 months	Converted to 3 dummy variables for urban, suburban, and rural (1 = yes, 0 = no)
	Single family home	Primary home dog has lived in the past 12 months is a single family home	1 = yes, 0 = no
	Water source	Primary address water source	Converted to 2 dummy variables for municipal water and well water (1 = yes, 0 = no)
	Central A/C	Primary address central A/C status	1 = yes, 0 = no
	Secondary home	Dog has a secondary address	1 = yes, 0 = no
Physical conditions	Height	Dog’s height in cm	Converted to 4 dummy variables based on quartiles: < 54.6, 54.6 - <57.2, 57.2 - <63.5, 63.5 and higher (1 = yes, 0 = no)
	Weight	Dog’s weight in kg	Converted to 4 dummy variables based on quartiles: <24.4, 24.4 - <27.7, 27.7 - <31.5, 31.5 and greater (1 = yes, 0 = no)
	BCS	Purina BCS	1, 2, 3,…9
	Parasite	Dog diagnosed with gastrointestinal parasite past 12 months	1 = yes, 0 = no
	Body system diagnosis	Whether the dog had any diagnoses in body system category	Converted to 9 dummy variables for ear/nose/throat, eye, gastrointestinal, infectious disease, musculoskeletal, reproductive, skin, urinary, and other (1 = yes, 0 = no)
Vaccines	Bordetella	Dog received Bordetella vaccine	1 = yes, 0 = no
	Rabies	Dog received rabies vaccine	1 = yes, 0 = no
	Other vaccine	Dog received a different vaccine than Bordetella and rabies	1 = yes, 0 = no
	Vaccine	Dog received any vaccine	1 = yes, 0 = no

### 2.2. Statistical analysis

Traditional regression models are prone to overfitting and inflated standard errors when a large number of predictors are used. Regularization methods, also called penalized regression models, reduce risk of overfitting by constraining the estimated coefficients and decreasing out of sample error. Elastic net models are a type of penalized regression technique that combines ridge regression and lasso regression. Elastic net models incorporate the ridge regression penalty which can handle correlated predictors and the feature selection characteristic of lasso regression (8). Elastic net logistic regression models were built with the ‘*elasticnet*’ package.

**Figure 1:**
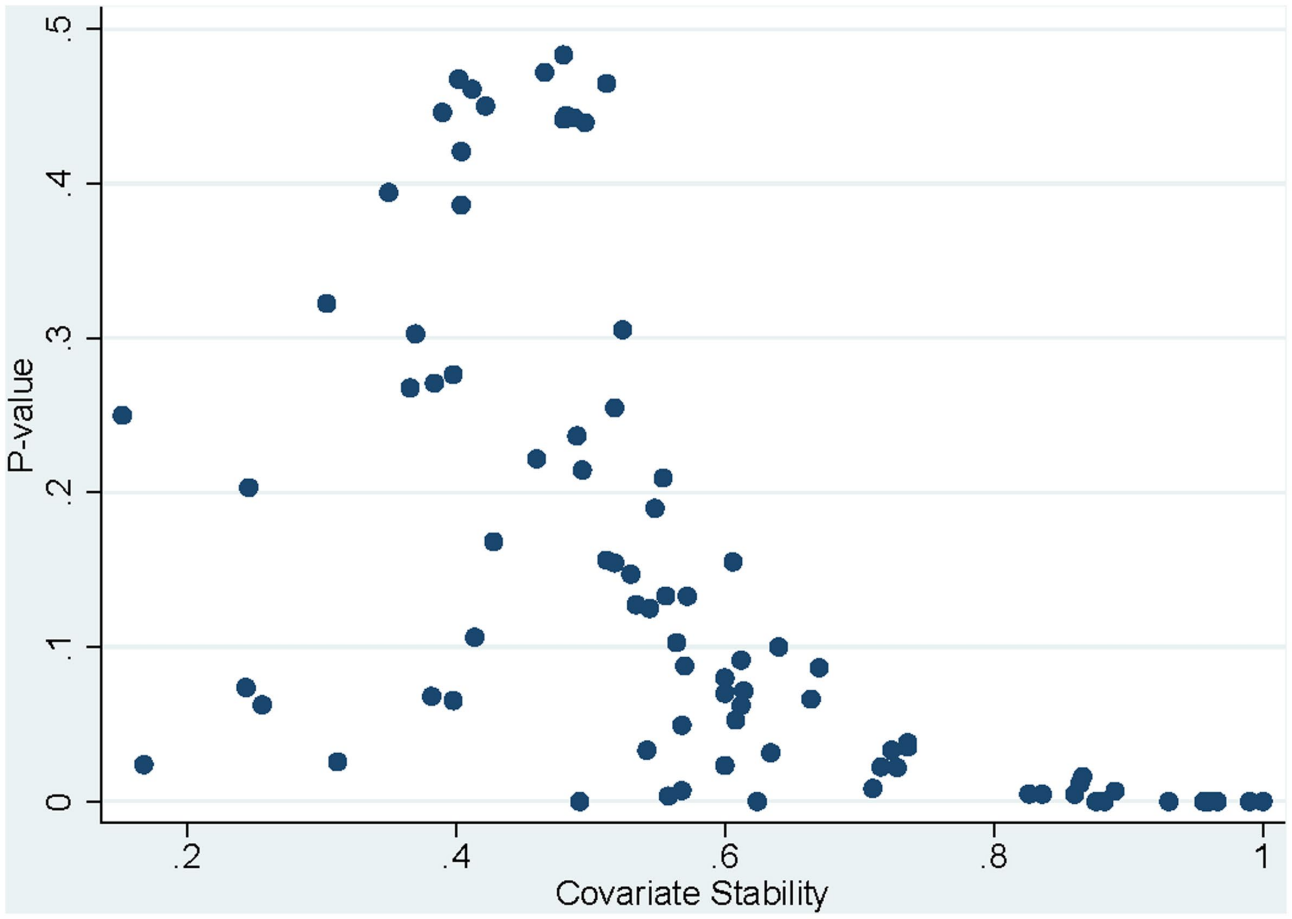
*p*-value versus covariate stability of variables in a bootstrapped elastic net model.

A bootstrapping procedure proposed by Bunea et al. (9) was used to calculate covariate stability. Bootstrapped value of ps and confidence intervals were calculated as previously reported for robust estimation of model parameters (10–12). This procedure involved performing 10-fold cross-validation 10 times in 500 bootstrapped samples, created using the *‘bsample’* command. The optimal tuning parameters, alpha and lambda, were selected through this process (8). A large range of alpha values was tested (0.1–1.0 at 0.1 intervals). A large range of lambdas was also tested using the ‘*selection* (cv.*, alllambdas*)’ option. Covariate stability was defined as the percent of time the covariate was selected to be in the model in the 500 bootstrapped samples. Bootstrapped value of ps were calculated as one minus the percent of time the coefficient estimates were on the majority side of zero. For example, if the coefficient estimate was greater than zero 450 out of 500 times, the bootstrapped value of *p* would be 1 – (450/500) = 0.10. Lastly, 90% bootstrapped confidence intervals were calculated using the ‘*bootstrap’* command. A plot of covariates stabilities against significance was used to determine thresholds for including variables in the final model. Variables were included in the final model if the bootstrapped value of *p* was <0.02 and the covariate stability was >80% (Figure 1) based on separation of the datapoints on the plot.

## 3. Results

### 3.1. Dataset description

The raw baseline dataset included data from 3,044 golden retrievers. After removing 46 records that had missing data in the final set of variables, 2,998 golden retrievers remained for analysis. Less than half (39.5%) of dogs were currently on heartworm preventative. The sample was approximately half female (49.5%) and male (50.5%). Less than half of males (32.9%) and females (42.0%) were neutered and spayed, respectively. Although age was not used in the analysis due to a high degree of missingness (29.7%), the mean age of the dogs in the 70.3% that reported age was 1.3 years, with a minimum and maximum value of approximately 5 months and 3 years, respectively. The American Heartworm Society recommends starting puppies on preventative as early as possible, no later than 8 weeks of age, so ideally all dogs in the sample would be on preventative.^3^ The sample represented the entire U.S., with 14.3% from the Pacific region, 27.8% from the South, 23.7% from the Midwest, 20.7% from the Northeast, and 13.5% from the Mountain region. The vast majority (83.1%) of dogs were considered companion animals. Supplementary Tables S1 and S2 describe heartworm preventative use stratified by predictor variables.

### 3.2. Elastic net model results

The final bootstrapped elastic net model is presented in Table 2. Dogs from the Southern U.S. had higher odds of being on heartworm preventative [OR (95% CI): 1.34 (1.33–1.35)] compared to other regions. Dogs from the Pacific region had lower odds [0.78 (0.77–0.79)] compared to other regions. There were no differences in heartworm preventative use in males (39.5%) versus females (39.7%), but altered dogs had higher heartworm preventative use (43.7%) compared to intact dogs (37.0%). In the final model, binary variables for neutered and intact males were retained. Neutered male dogs had higher odds of being on heartworm preventative [1.19 (1.18–1.20)] compared to the other sex categories, while intact male dogs had lower odds [0.859 (0.854–0.865)]. Dogs in the highest height quartile (63.5 cm and taller) had lower odds [0.742 (0.735–0.750)] of being on heartworm preventative compared to shorter dogs.

**Table 2:** Factors associated with heartworm preventative use in a sample of golden retrievers using a bootstrapped elastic net model (*N* = 2,998 dogs).

Variable^1^	*n* (number in variable category)	Covariate Stability^2^	Bootstrapped OR^3^	Lower 90% bootstrapped CI	Upper 90% bootstrapped CI	Bootstrapped *p*-value^4^
Intercept	–	1.00	0.167	0.162	0.170	0.0000
Region (South)	832	0.99	1.34	1.33	1.35	0.0000
Region (Pacific)	428	0.93	0.78	0.77	0.79	0.0000
Sex (male neutered)	498	0.88	1.19	1.18	1.20	0.0000
Sex (male intact)	1,016	0.88	0.859	0.854	0.865	0.0000
Height (63.5 cm and taller)	326	0.97	0.742	0.735	0.750	0.0000
Infectious disease system diagnosis	714	0.84	1.15	1.14	1.16	0.0048
Ear/nose/throat system diagnosis	594	0.89	1.17	1.16	1.18	0.0067
Sun exposure location (concrete)	442	0.86	1.19	1.18	1.20	0.0047
Prior heartworm preventative use	160	0.96	1.47	1.45	1.49	0.0000
Supplements given	1,460	0.87	0.889	0.884	0.894	0.0162
On tick preventative	351	1.00	20.7	20.4	21.1	0.0000
# of rooms with carpeted flooring	N/A^5^	0.86	1.026	1.025	1.027	0.0116
Spends time indoors in a room with hardwood floor	1,620	0.96	1.20	1.19	1.21	0.0000
Received Bordetella vaccine	1,439	1.00	1.40	1.39	1.41	0.0000
Received rabies vaccine	2,505	0.83	1.24	1.23	1.26	0.0048
Received a different vaccine than rabies and Bordetella	2,563	0.99	1.60	1.58	1.62	0.0000

1 Variables were included in the final model if the bootstrapped *p*-value was < 0.02 and the covariate stability was > 80% (Figure 1).

2 Covariate stability was defined as the percent of time the covariate was selected to be in the model in 500 bootstrapped samples.

3 Bootstrapped ORs and 90% CI calculated from 500 bootstrapped samples. Each variable that is a level of a categorical variable is being compared to all other levels of that same categorical variable.

4 Bootstrapped value of ps were calculated as one minus the percent of time the coefficient estimates were on the majority side of zero.

5 Numerical predictor. Median 3, minimum 0, maximum 14.

Dogs with an infectious disease diagnosis or an ear/nose/throat system disease diagnosis had higher odds of being on heartworm preventative [1.15 (1.14–1.16) and 1.17 (1.16–1.18), respectively]. Those that had prior heartworm preventative use had almost 50% greater odds [1.47 (1.45–1.49)] of currently being on heartworm preventatives. Interestingly, those that were on supplements had lower odds [0.889 (0.884–0.894)] of being on heartworm preventative. The variable most strongly associated with heartworm preventative use was being on tick preventative. Those that were on tick preventative had 20.7 times greater odds (95% CI: 20.4–21.1) of being on heartworm preventative compared to those not on tick preventative.

Being exposed to sun on concrete flooring was associated with higher heartworm preventative use [1.19 (1.18–1.20)]. The odds of being on heartworm preventative increased with the number of carpeted rooms in the house [1.026 (1.025–1.027)]. Spending time indoors on a room with hardwood flooring was associated with greater odds [1.20 (1.19–1.21)] of being on heartworm preventative.

Dogs that received vaccines had higher odds of being on heartworm preventative, with the highest odds [1.60 (1.58–1.62)] among those that received a different vaccine other than rabies and Bordetella (dogs in this category could also have gotten rabies and/or Bordetella vaccinations).

## 4. Discussion

There are very few studies that have investigated what factors influence heartworm preventative use in dogs (5, 6). To address this knowledge gap, we examined multiple factors associated with heartworm preventative use in a large sample of golden retrievers from the Golden Retriever Lifetime Study (7). Due to the high number of predictors, we implemented a robust statistical analysis technique (bootstrapped elastic net regression) that can handle multicollinearity unlike traditional regression techniques. Regularization methods, also called penalized regression models, reduce risk of overfitting by constraining the estimated coefficients and decreasing out of sample error (13). We identified multiple variables associated with heartworm preventative use, including vaccine use, U.S. region, sex, height, other disease conditions, medication and supplementation history, and characteristics of the spaces where the dog spends time.

Overall, the prevalence of heartworm preventative use was 39.5%. This was much lower than an estimate from a sample of dogs presenting at the veterinary teaching hospital at the University of Pennsylvania, which reported that 79.8% of dogs were currently on preventatives at the time of their admittance (5). In our study, even among dogs located in the Southern U.S., which has the highest prevalence of heartworm preventative use and rate of heartworm disease, prevalence was only 45.9% (2, 6). Correspondingly, use of heartworm preventatives was lowest in the Pacific region which has some of the lowest rates of heartworm disease (2). Our estimates are similar to results reported in Bir et al., 2018 which found that 54% used heartworm prevention in a nationally representative sample. We also found that dogs in the highest quartile of height were less likely to be on heartworm preventative. Height is likely correlated with weight and cost could be prohibitive (14). However, weight did not make it into the final model, so the mechanism is unclear. It could be due to the distribution of the data and the quartile ranges may not correspond directly to the weight ranges of heartworm medications. We also found that sex was associated with heartworm preventative use. The “sex” variable was a combination of biological sex and spay/neuter status. While there were no differences between males versus females, intact males and females had lower rates of heartworm preventative use compared to their altered counterparts. Spay/neuter status likely represents owner investment in health and veterinary care. This corroborates other research from Gates and Nolan (5) who found that intact dogs were less likely to be on preventative compared to neutered dogs and that overall, there were no differences between males and females.

Dogs that received a Bordetella vaccine, rabies, or other vaccine had greater heartworm preventative use. This could be due to greater trust in veterinarian recommendations (15). Interestingly, supplements were associated with lower heartworm preventative use. Pet owners that use supplements may prefer a holistic, naturopathic approach to pet care. They may use supplements such as black walnut or home remedies such as brewer’s yeast or essential oils instead of heartworm preventatives (15, 16). Prior use of heartworm preventatives and current use of tick preventatives were strongly associated with current heartworm preventative use which is not surprising since some heartworm preventatives also protect against infections from ticks. Offering a combination drug may increase adherence to heartworm preventatives, especially if owners do not understand and perceive the risk of heartworm disease (17). Dogs that were diagnosed with an infectious disease system or an ear/nose/throat system disease were more likely to be on preventative. The infectious disease system includes gastrointestinal parasites; therefore, owners may be more likely to be on medications for parasite control after a diagnosis and many of these medications include heartworm control. The mechanism behind why an ear/nose/throat system diagnosis is associated with greater heartworm preventative use is unclear. One possible mechanism is that some of the symptoms of heartworm disease involve the ear/nose/throat system, such as coughing, so the dog may have been tested for heartworm disease and then subsequently put on preventative. In a survey among members of a hunting club, only 70% tested annually, which is expected to be lower in the general population (17). Some of the dogs that do not get tested annually may only be tested when ill and presenting with symptoms that could be attributed to heartworm disease.

Dogs that were exposed to the sun on concrete flooring were more likely to be on preventative. The other sun exposure flooring categories were grass and dirt but were not retained in the final model during the variable selection process. Concrete flooring may be significant as it may indicate a closer proximity of the dog to the house, whereas dogs kept on grass and dirt may include dogs that free roam and that receive poorer general care compared to confined dogs (18). Heartworm preventative use increased with the number of carpeted rooms in the house and was higher among dogs that spent time on hardwood floors. Both variables could be proxies for socioeconomic status. Owners of higher socioeconomic status may be more likely to spend money on heartworm preventative as they have more disposable income. However, Gates and Nolan (5) did not find that income was significantly associated with heartworm preventative use in multivariable analyses, although Evason et al. (15) reported that 33% of clients said cost was a barrier and downside to using heartworm preventatives. It is of note that variables that represent attachment and the human-animal bond were not significant in the final model. This is similar to results reported in Shore et al. (19) who did not find a clear trend between attachment levels and heartworm preventative use.

A major limitation of this study is the limited generalizability outside the study population. The study population consisted of all golden retrievers that were less than 6 years of old. The results of the study may not be generalizable to other breeds, older dogs, and to the general dog population. In addition, owners that participate in the GRLS may be inherently different than owners that do not participate. Owners that participate may be more invested in their dog therefore more likely to adopt veterinarian recommendations for heartworm prevention. However, this does not seem to be the case, as the prevalence of heartworm preventative use was low in our sample and was close to other national estimates (6). Another limitation is that we did not have owner demographic data. We were only able to infer socioeconomic status from some of the variables. Owner factors including gender, age, and socioeconomic status have been shown to be associated with heartworm preventative use (14). This study also had many strengths. We were able to leverage a large dataset representative of the U.S. that collected multiple variables in a standardized questionnaire. In addition, we were able to evaluate a number of factors that have not been tested for their relationship with heartworm preventative use prior to this study. Still, the findings of this study can be strengthened by conducting additional studies outside of the study population.

Overall, we found multiple factors associated with heartworm preventive use. Results of this study can be used to help veterinarians understand motivations and factors behind heartworm preventative use in dogs so that client communication can be improved. Results can also be used to identify populations at risk of low heartworm preventative use so that education and outreach efforts can be better targeted. Future studies can confirm the findings in a more diverse study population and use the results of this study to develop and test client communication and educational interventions.

## Data availability statement

The data analyzed in this study is subject to the following licenses/restrictions: Data can be requested through the Morris Animal Foundation Golden Retriever Lifetime Study RFP. Requests to access these datasets should be directed to https://www.morrisanimalfoundation.org/golden-retriever-lifetime-study-rfp.

## Ethics statement

The secondary data analysis conducted in this study was approved by the Lincoln Memorial University Institutional Review Board.

## Author contributions

LW was involved in developing the project proposal, study design, data analysis, and writing the first draft of the manuscript. CF and VF were involved with study design and editing the manuscript. All authors contributed to the article and approved the submitted version.

## Funding

This study was supported by the Golden Retriever Lifetime Study and this manuscript were made possible through financial support provided by the Morris Family Foundation, the Mark & Bette Morris Family Foundation, VCA, the V Foundation, Blue Buffalo Company, Petco Love, Zoetis, Antech Inc., Elanco, the Purina Institute, Orvis, the Golden Retriever Foundation, the Hadley and Marion Stuart Foundation, Mars Veterinary, generous private donors and the Flint Animal Cancer Center at Colorado State University. The funders had no role in study design, data collection and analysis, decision to publish, or preparation of the manuscript.

## Conflict of interest

The authors declare that the research was conducted in the absence of any commercial or financial relationships that could be construed as a potential conflict of interest.

## Publisher’s note

All claims expressed in this article are solely those of the authors and do not necessarily represent those of their affiliated organizations, or those of the publisher, the editors and the reviewers. Any product that may be evaluated in this article, or claim that may be made by its manufacturer, is not guaranteed or endorsed by the publisher.

## Footnotes

^1^ https://www.morrisanimalfoundation.org/sites/default/files/filesync/GRLS-Annual-Veterinarian-Questionnaire.pdf

^2^ https://www.morrisanimalfoundation.org/sites/default/files/filesync/GRLS-Annual-Owner-Questionnaire.pdf

^3^ https://www.heartwormsociety.org/images/pdf/2018-AHS-Canine-Guidelines.pdf
